# Regulatory Factor X1 Downregulation Contributes to Monocyte Chemoattractant Protein-1 Overexpression in CD14+ Monocytes *via* Epigenetic Mechanisms in Coronary Heart Disease

**DOI:** 10.3389/fgene.2019.01098

**Published:** 2019-11-01

**Authors:** Sujie Jia, Shuang Yang, Pei Du, Keqin Gao, Yu Cao, Baige Yao, Ren Guo, Ming Zhao

**Affiliations:** ^1^Department of Pharmacy, The Third Xiangya Hospital, Central South University, Changsha, China; ^2^Center of Clinical Pharmacology, The Third Xiangya Hospital, Central South University, Changsha, China; ^3^Department of Pharmacy, Weifang People’s Hospital, Weifang, China; ^4^Dapartment of Cardiology, The Third Xiangya Hospital, Central South University, Changsha, China; ^5^Dapartment of Dermatology, Hunan Key Laboratory of Medical Epigenomics, The Second Xiangya Hospital, Central South University, Changsha, China

**Keywords:** monocytes, histone acetylation, monocyte chemoattractant protein-1, regulatory factor X1, epigenetics

## Abstract

Monocyte chemoattractant protein 1 (MCP1) affects the chemotaxis of monocytes and is a key chemokine closely related to the development of atherosclerosis (AS). Compared with healthy controls, coronary heart disease (CAD) patients show significantly upregulated plasma concentrations and mRNA expression of MCP1 in CD14+ monocytes. However, the specific regulatory mechanism of MCP1 overexpression in AS is still unclear. Our previous research indicated that there was no significant difference in the H3K4 and H3K27 tri-methylation of the *MCP1* promoter in CD14+ monocytes from CAD *versus* non-CAD patients, but the H3 and H4 acetylation of the *MCP1* promoter was increased in CD14+ monocytes from CAD patients. We further found that the H3K9 tri-methylation of the *MCP1* promoter in CD14+ monocytes from CAD patients was decreased, but the DNA methylation levels did not differ markedly from those in non-CAD patients. Our previous work showed that the level of regulatory factor X1 (RFX1) was markedly reduced in CD14+ monocytes from CAD patients and played an important role in the progression of AS by regulating epigenetic modification. In this study, we investigated whether RFX1 and epigenetic modifications mediated by RFX1 contribute to the overexpression of MCP1 in activated monocytes in CAD patients. We found that the enrichment of RFX1, histone deacetylase 1 (HDAC1), and suppressor of variegation 3–9 homolog 1 (SUV39H1) in the *MCP1* gene promoter region were decreased in CD14+ monocytes from CAD patients and in healthy CD14+ monocytes treated with low-density lipoprotein (LDL). Chromatin immunoprecipitation (ChIP) assays identified *MCP1* as a target gene of RFX1. Overexpression of RFX1 increased the recruitments of HDAC1 and SUV39H1 and inhibited the expression of MCP1 in CD14+ monocytes. In contrast, knockdown of RFX1 in CD14+ monocytes reduced the recruitments of HDAC1 and SUV39H1 in the *MCP1* promoter region, thereby facilitating H3 and H4 acetylation and H3K9 tri-methylation in this region. In conclusion, our results indicated that RFX1 expression deficiency in CD14+ monocytes from CAD patients contributed to MCP1 overexpression *via* a deficiency of recruitments of HDAC1 and SUV39H1 in the *MCP1* promoter, which highlighted the vital role of RFX1 in the pathogenesis of CAD.

## Introduction

Coronary heart disease (CAD) is the predominant cause of mortality worldwide (Chistiakov et al., 2016). Atherosclerosis (AS) is the main pathological basis of CAD, and its pathogenesis has not been fully elucidated thus far. A large number of basic and clinical studies have shown that AS and CAD secondary to AS involve a large number of chronic inflammatory reactions (Annika et al., 2007; Hohensinner et al., 2011; Kolattukudy et al., 2012). Monocytes, the main components of the innate immune system, play an important role in the initiation and development of AS (Ley et al., 2011). Monocytes are not only effector cells of inflammatory factors and adhesion molecules, but also secrete cellular inflammatory factors such as interleukin-6 (IL-6), interleukin-1 (IL-1) and tumor necrosis factor-α (TNF-α) which can aggravate the inflammatory response (Ghattas et al., 2013).

Monocyte chemoattractant protein-1 (MCP1), also known as CCL2, is a member of the β family of the chemotactic cytokine family that can recruit monocytes to infiltrate the arterial wall and promote adhesion between leukocytes and endothelial cells (Kuziel et al., 1997; Boisvert et al., 2000). Numerous studies have demonstrated that increased expression of MCP1 is closely related to the development of AS both *in vivo* and *in vitro*. Inhibition of MCP1 reduces monocyte adhesion and decreases lesion size both *in vitro* and in mice that overexpress human apolipoprotein B (Gosling et al., 1999). More importantly, monocytes may be involved in the amplification of their own recruitment to inflammatory lesions by inducing MCP1 (Cushing and Fogelman, 1992). A previous study also showed a significant increase in MCP1 expression in CAD patients and LDL-treated monocytes (Du et al., 2019). However, the specific regulatory mechanisms of MCP1 overexpression in CD14+ monocytes are not fully understood.

Recent studies have shown that abnormal epigenetic modification plays an important role in the pathogenesis of AS (Du et al., 2019). In apoE^-/-^ mouse aortic plaques and peritoneal macrophages, hypermethylation of the cystathionine-gamma lyase (*CSE*) promoter region resulted in a reduction in *CSE* gene expression, thereby promoting AS development (Du et al., 2016). Another study found that DNA methylation and histone H3K9 and H3K27 methylation levels were significantly shown in human carotid atherosclerotic plaques (Greißel et al., 2015). Our previous research indicated that histone acetylation of the *MCP1* gene promoter was elevated in CD14+ monocytes from CAD patients, but H3K4 and H3K27 tri-methylation showed no difference between CAD and non-CAD controls (Xiao et al., 2018). However, whether MCP1 overexpression in CD14+ monocytes from CAD patients is due to the adaptation of H3K9 tri-methylation and DNA methylation levels in the promoter region is not known.

LDL is an important risk factor for AS. The levels of ox-LDL and small dense LDL (sdLDL) in peripheral blood from patients with CAD were observed to be significantly higher than those in healthy controls (Tenjin et al., 2014). In addition to promoting the differentiation of monocytes into macrophages, LDL also functions in promoting AS by enhancing monocyte adhesion, injuring vascular endothelial cells, and promoting foam cell formation (Escate et al., 2016).Ox-LDL promotes monocyte activation and this effect is closely related to the induction of MCP1 (Feng, Y. et al., 2014; Zidar et al., 2015). Studies have also shown that the atherogenic effect of LDL is associated with epigenetic modification. DNA methylation, histone modification, and micro-RNA are all associated with atherogenic effects of LDL (Chen et al., 2012; Zhang and Wu, 2013). Ox-LDL inhibits the methylation of the *CSE* gene promoter region in mouse macrophages, which in turn activates macrophage inflammatory responses (Du et al., 2016). The mechanism whereby LDL regulates MCP1 expression in CD14+ monocytes is still unclear.

The regulatory factor X (RFX) family was first discovered in mammals approximately 20 years ago and is evolutionarily conserved; these proteins contain 76 highly conserved amino acid sequences, have the appearance of winged helix proteins, and have the ability to combine with a cis-acting element X box (Emery et al., 1996). Previous studies have shown that RFX1 is significantly downregulated in tumors such as gliomas and autoimmune diseases such as systemic lupus erythematosus (Ohashi et al., 2004; Cheng et al., 2016; Zhao et al., 2010a. RFX1 mediated dimerization and transcriptional repression functions by recruiting epigenetic enzymes such as DNA methyltransferase 1 (DNMT1), histone deacetylase 1 (HDAC1), and histone-lysine N-methyltransferase SUV39H1 (SUV39H1) (Katan-Khaykovich and Shaul, 1998). RFX1 downregulation causes CD11a, CD70 and IL17A overexpression by reducing DNA methylation and increasing H3 acetylation levels in the promoter region of CD11a, CD70 and IL17A in the CD4+ T cells of systemic lupus erythematosus (SLE) patients, which contributes to autoimmune responses (Zhao et al., 2010a; Zhao et al., 2018). Our previous research also showed that RFX1 expression was downregulated in the CD14+ monocytes of CAD patients (Du et al., 2019).

In this study, we found that overexpression of MCP1 in CD14+ monocytes from CAD patients decreased levels of H3K9 tri-methylation in the *MCP1* promoter region, rather than DNA methylation. We further identified that downregulation of RFX1 led to overexpression of the target gene *MCP1* through the recruitment of HDAC1 and SUV39H1 to regulate histone modifications in the *MCP1* promoter. In addition, LDL promoted MCP1 expression by regulating the recruitment of RFX1 and its related epigenetic modification enzymes in the *MCP1* promoter region of CD14+ monocytes. These findings demonstrate the role and mechanism of RFX1 in regulating MCP1 expression in CAD patients, which suggests the occurrence of a novel epigenetic mechanism in AS development.

## Materials and Methods

### Subject

CAD patients were diagnosed by positive coronary angiography (at least one coronary artery stenosis with a stenosis ≥50% of the diameter) and non-CAD patients (confirmed with negative coronary angiography results) were recruited from the Department of Cardiology of Xiangya Third Hospital. None of the patients were treated with PTCA or stenting. Subjects with valvular heart disease, acute infection, severe liver, and kidney dysfunction or a history of using statins within 8 weeks were excluded. All candidates gave informed consent. This study was approved by the Ethics Committee of the Third Xiangya Hospital of Central South University. The characteristics of the patients were shown in **Table S1** in the **Supplementary Material**.

### Isolation of Peripheral Blood CD14+ Monocytes

Peripheral blood mononuclear cells (PBMCs) were isolated by Ficoll-Hypaque density gradient centrifugation (GE, USA). CD14+ monocytes were then isolated from the PBMCs by positive selection using magnetic beads (Miltenyi Biotec, Germany) according to the protocol provided by the manufacturer.

### Cell Culture

All CD14+ monocytes were cultured in RPMI 1640 medium (Gibco, USA) supplemented with 10% fetal bovine serum (Capricorn Scientific, USA). Human umbilical vein endothelial cells (HUVECs) were cultured with Dulbecco’s Modified Eagle Medium (DMEM) basic (1×) (Gibco, USA) that contained 10% fetal bovine serum. The cultures were incubated at 37°C in a humidified atmosphere containing 5% CO_2_.

### Enzyme Linked Immunosorbent Assay

Human MCP1 Quantikine ELISA Kits (Senxiong Biological, China) were used. All procedures were performed according to the manufacturer’s instructions.

### RNA Extraction and Real-Time Quantitative Polymerase Chain Reaction

Total RNA was isolated from CD14+ monocyte cells with TRIzol Reagent (Invitrogen, USA) according to the manufacturer’s instructions, and stored at -80°C for further use. RT-qPCR was performed with a LightCycler 96 (Roche, Switzerland). mRNA levels were quantified using a SYBR Prime Script RT-PCR kit (Takara, Japan). β-Actin was amplified as an endogenous control. All reactions were run in triplicate. The sequences of the synthetic oligonucleotides used as primers are shown in **Table S2** in the **Supplementary Material**.

### Pyrophosphate Sequencing

The genomic DNA was extracted and treated with bisulfite. And then the promoter fragment of MCP1 gene was amplified by PCR using the treated genomic DNA. The product of PCR was sequenced by PyroMark Q96 sequencer. The primer sequences and related information are shown in **Table S3** in the **Supplementary Material**.

### Chromatin Immunoprecipitation Assay-Quantitative Polymerase Chain Reaction

Chromatin Immunoprecipitation Assay (ChIP) analysis was performed with a ChIP assay kit (Millipore, USA) according to the instructions. In brief, CD14+ monocyte cells were fixed for 8 min at room temperature with 1% formaldehyde. Glycine was then added to a final concentration of 0.125 M to quench the formaldehyde. The monocytes were pelleted, washed once with ice-cold phosphate buffered saline (PBS), and lysed. The lysates were pelleted, resuspended, and sonicated to reduce DNA to 200 to 1,000 bp fragments. Chromatin was precipitated with protein A agarose beads for 1 h and then incubated with tri-methylated H3K9 antibody (Abcam, USA), anti-histone H3/H4 acetylation antibody (Millipore, Germany), anti-RFX1 antibody (Santa Cruz), anti-DNMT1 antibody (Abcam, USA), anti-HDAC1 antibody (Abcam, USA), anti-SUV39H1antibody (Abcam, USA) or control mouse IgG (Millipore, Germany) overnight. The immunocomplexes were further precipitated with protein A agarose beads, washed, and eluted in 100 ml of TE with 0.5% SDS and 200 mg/ml proteinase K. Precipitated DNA was further purified through phenol/chloroform extraction and ethanol. The fold enrichment was quantified by using qPCR and calculated relative to the respective input DNA. The primers used are shown in **Table S4** in the **Supplementary Material**.

### Lentivirus Infection

The isolated CD14+ monocytes incubated with or without LDL at 100 mg/L for 24 h were randomly divided into two groups. CD14+ monocytes were infected respectively with lentivirus containing RFX1-overexpressing vector, lentivirus containing empty control vector, lentivirus containing RFX1-shRNA expression vector and lentivirus containing negative control vector which were purchased from Shanghai Jikai Gene Co., Ltd. The appropriate amount of lentivirus was obtained according to the multiplicity of infection (MOI = 5). The lentivirus mixture was added to the cell suspension and mixed by gentle pipetting. After 72 h, the infection efficiency was observed by fluorescence inverted microscopy. Cells were harvested for subsequent experiments after the confirmation of successful infection.

### Western Blotting

The concentration of protein lysate obtained from CD14+ monocyte was determined by BCA™ Protein assay kits (Pierce, USA). The 30 µg protein taken from each sample was denatured at 99°C for 7 min. Proteins were separated by SDS-PAGE gel electrophoresis and then transferred to a PVDF membrane. The PVDF membrane was incubated overnight with RFX1 antibody (Genetex, USA) and beta-actin antibody diluted at a volume ratio of 1:1,000 (Santa Cruz, USA). After chemiluminescence, the image gray value of each image was analyzed by Image pro plus 6.0 software. The β-actin protein was used as the internal reference, and the ratio of the gray value of RFX1 protein to β-actin protein was used as the RFX1 protein relative expression.

### Statistical Analysis

Data were analyzed using SPSS 19.0 software (SPSS, USA). The measurement data were expressed as the mean ± standard deviation (mean ± SEM). The normal distributed data were compared by using *t*-tests. *P* < 0.05 was considered significant.

## Results

### Expression and Epigenetic Modifications of Monocyte Chemoattractant Protein-1 in CD14+ Monocytes From Coronary Heart Disease Patients

**Figure 1:**
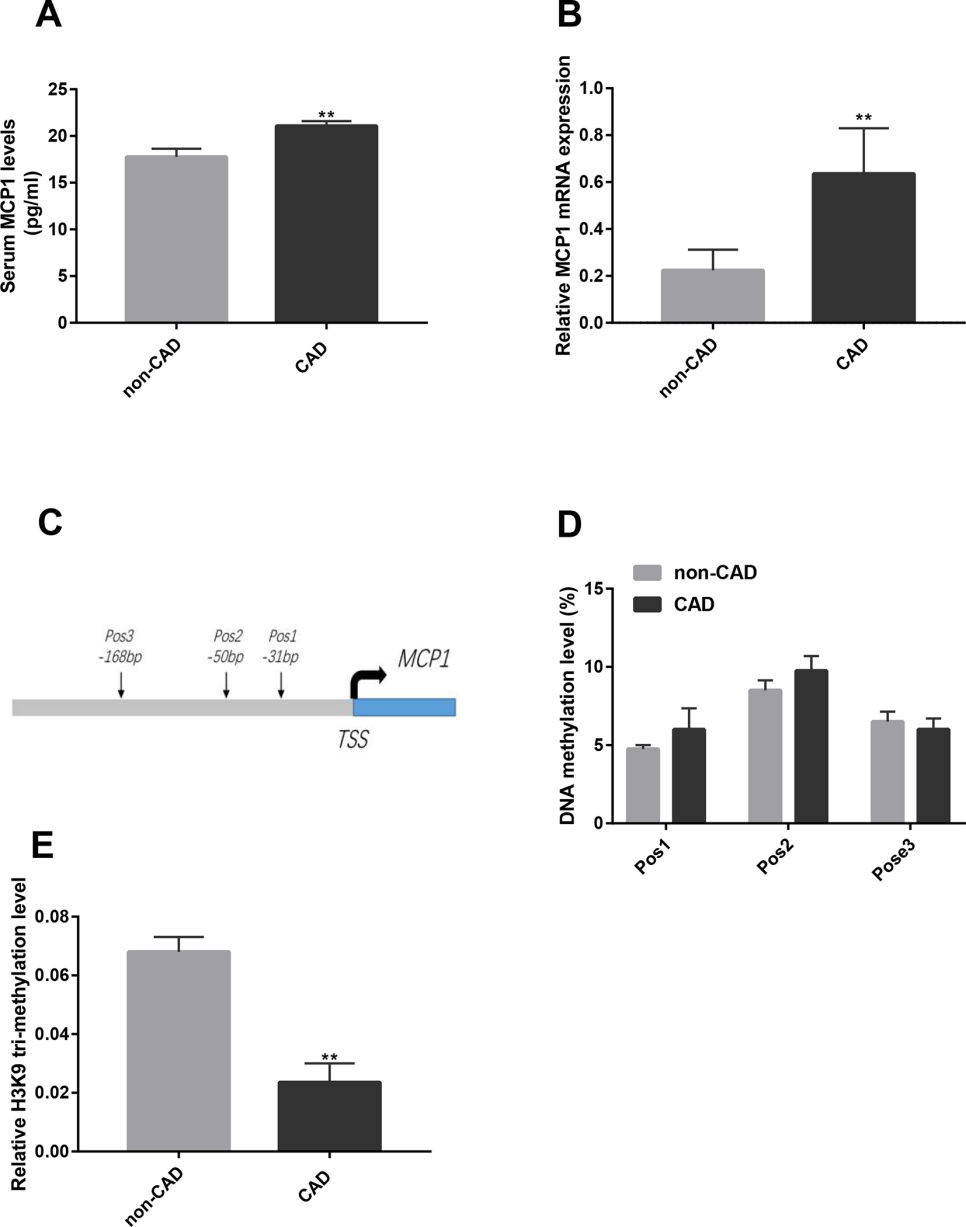
Expression and epigenetic modifications of *MCP1* gene in CD14+ monocytes from CAD patients. **(A)** The concentration of MCP1 in the plasma of CAD (n = 13) and non-CAD (n = 8) patients was detected by ELISA. **(B)** The mRNA expression of MCP1 in CD14+ monocytes from CAD (n = 17) and non-CAD (n = 19) patients was determined by RT-qPCR. **(C)** A genomic diagram to indicate the genomic locations of CG pairs detected by pyro-sequencing. **(D)** The mean methylation level of the *MCP1* gene promoter in CD14+ monocytes from CAD patients and non-CAD controls was measured by bisulfite sequencing (n = 4). **(E)** ChIP-qPCR analysis was used to detect relative histone H3K9 tri-methylation level of the *MCP1* gene promoter in CD14+ monocytes from CAD patients and non-CAD controls (n = 3). The data shown are the mean ± SEM. **P 0.01 relative to the control.

To investigate the difference in MCP1 expression between CAD patients and non-CAD patients, we analyzed the expression of MCP1 in plasma and MCP1 mRNA expression in CD14+ monocytes from two groups. As shown in **Figures 1A–B**, the expression level of MCP1 in the plasma of CAD patients and MCP1 mRNA expression in CD14+ monocytes from CAD patients were significantly higher than in non-CAD controls.

Studies have found that the epigenetic modification status of the promoter region of the *MCP-1* gene can affect MCP1 expression (Peng et al., 2015; Lin et al., 2017). Our previous study also showed that the levels of H3 and H4 acetylation in the promoter region of the *MCP1* gene in CD14+ monocytes was significantly higher in patients with CAD than in non-CAD patients (Xiao et al., 2018). Here, we examined the DNA methylation and histone modification of the promoter region of *MCP1* in CD14+ monocytes from CAD and non-CAD patients. The results of pyrophosphate sequencing (pyro-sequencing) showed that the methylation level has no significant difference in each CpG site (-31 bp, -50 bp and -168 bp) upstream of transcription starting site of *MCP1* gene in CD14+ monocytes from patients with CAD *versus* non-CAD controls (**Figures 1C, D**). However, ChIP-qPCR result indicated that the relative level of histone H3K9 tri-methylation (**Figure 1E**) in the promoter region of the *MCP1* gene in CD14+ monocytes from CAD patients was significantly lower than in those from non-CAD patients.

### Regulatory Factor X1 Binds to the Promoter Region of Monocyte Chemoattractant Protein-1 Gene

**Figure 2:**
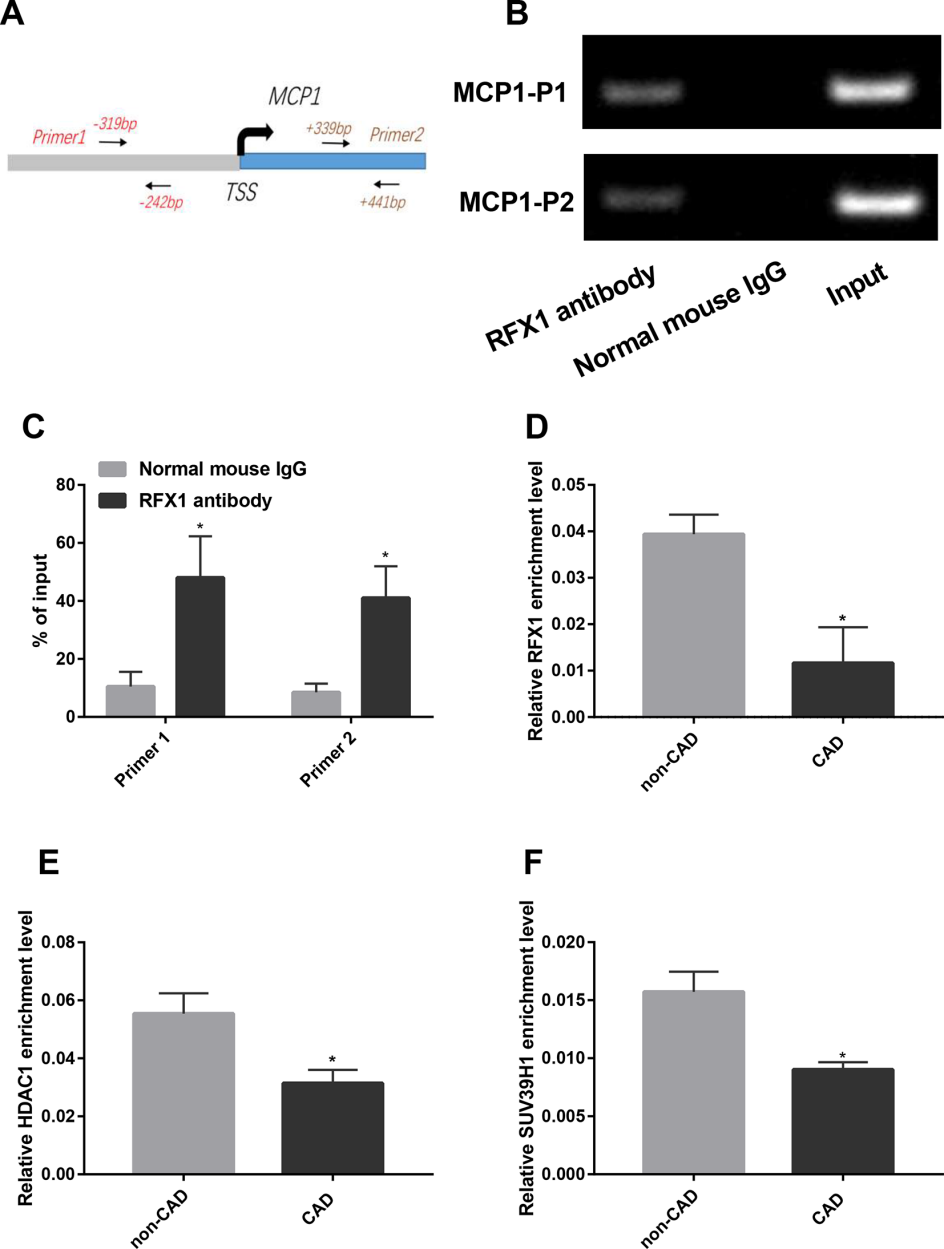
RFX1 binds to the promoter region of *MCP*. **(A)** A genomic diagram to indicate the genomic locations of primers used in ChIP-PCR**. (B)** Band diagram of normal PCR amplification of the ChIP product in CD14+ monocyte. **(C)** Statistical results of band densities indicate the binding of RFX1 to *MCP1* promoter determined by ChIP-PCR in CD14+ monocyte. **(D**–**F)** Relative recruitment levels of RFX1 **(D)**, HDAC1 **(E)** and SUV39H1 **(F)** in the promoter region of *MCP1* were detected by ChIP-qPCR in CD14+ monocytes from CAD and non-CAD patients. All values were the average of at least three biological replicates, and the data shown are mean ± SEM. *P 0.05 relative to the control.

We used ChIP-PCR to verify whether RFX1 binds the promoter region of the *MCP1* gene in CD14+ monocytes. The primers for ChIP-PCR were shown in **Figure 2A**. As shown in **Figure 2B**, the product amplified from the input DNA without RFX1 antibody precipitation exhibited the strongest electrophoresis band, while the normal mouse IgG group (negative antibody control) showed no specific amplified fragment after PCR. The amplified fragments of the promoter region of the *MCP1* gene were visualized by using DNA fragments that specifically bind to the RFX1 antibody after amplification by PCR. The comparison of band densities indicated that RFX1 can bind in the promoter region of the *MCP1* gene (*P* < 0.05) (**Figure 2C**).

Previous studies showed that RFX1 bound to target gene promoter region and regulated its epigenetic modification (Du et al., 2019; Zhao et al., 2010b). We further compared the enrichments of RFX1 and its related modification enzymes in *MCP1* promoter region between CAD and non-CAD patients. ChIP-qPCR results suggested that the recruitment of RFX1, HDAC1 and SUV39H1 in the promoter region of the *MCP1* gene in CAD CD14+ monocytes was significantly lower than that in the control group (**Figures 2D–F**).

### Low-Density Lipoprotein Altered Histone Modifications of the Monocyte Chemoattractant Protein-1 Promoter Region in CD14+ Monocytes

**Figure 3:**
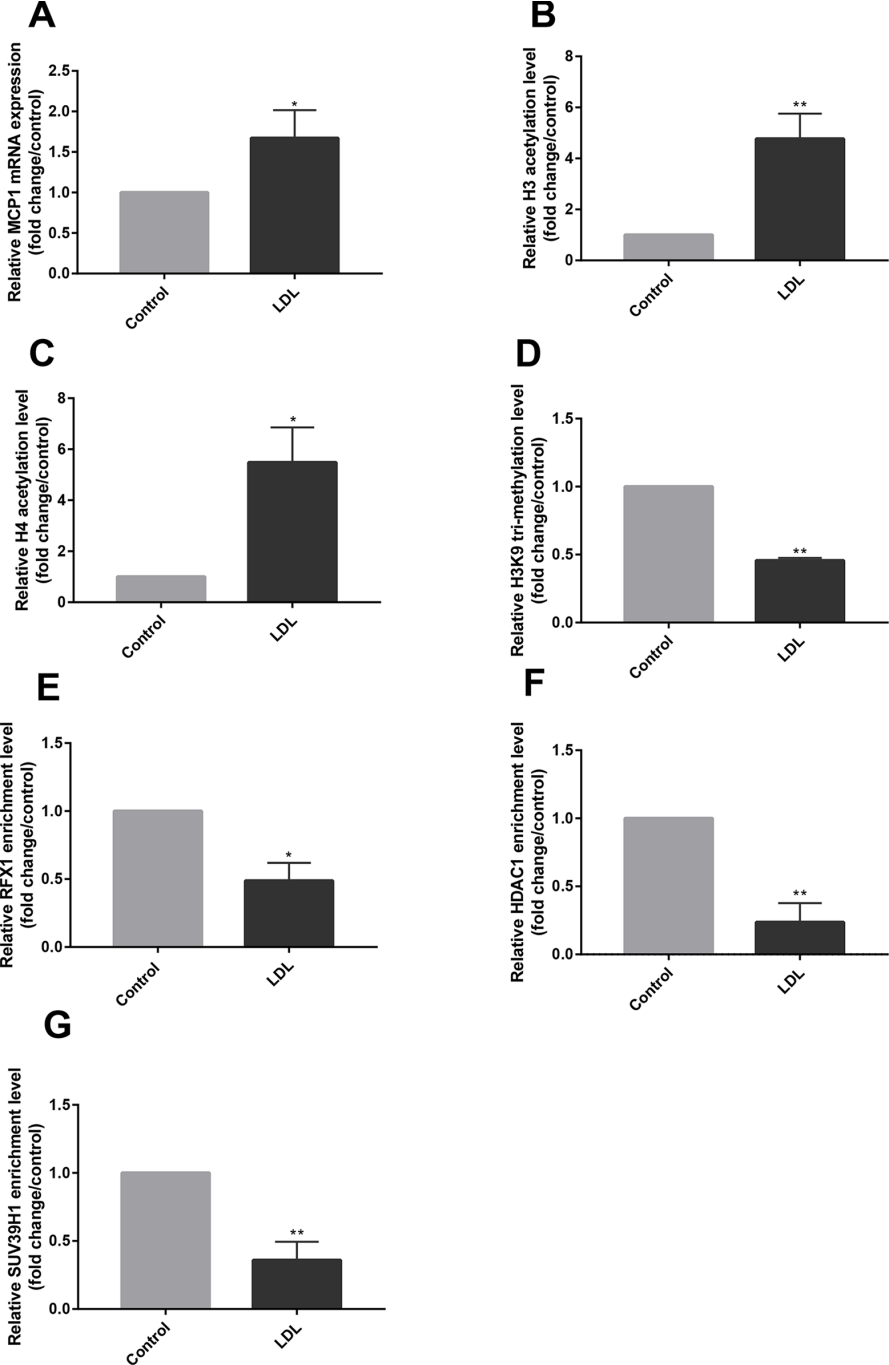
LDL altered histone modifications of the *MCP1* promoter region in CD14+ monocytes. CD14+ monocytes were treated with LDL at 100 mg/L for 24 h. **(A)** The relative mRNA expression of MCP1 in CD14+ monocytes was detected by RT-qPCR. **(B**–**D)** ChIP-qPCR was used to measure the fold change of relative histone H3 **(B)** and H4 acetylation **(C)** and H3K9 tri-methylation **(D)** in the promoter region of the *MCP1* gene in CD14+ monocytes treated with or without LDL. **(E–G)** The fold change of relative recruitments of RFX1 **(E)**, HDAC1 **(F)** and SUV39H1 **(G)** in the promoter region of the *MCP1* gene in CD14+ monocytes were detected by ChIP-qPCR. All values are the average of at least three biological replicates, and the data shown are the mean ± SEM. *P 0.05 and **P 0.01 compared with the control group.

The CD14+ monocytes isolated from healthy human peripheral blood were incubated with LDL for 24 h to detect the effect of LDL. RT-qPCR results showed that LDL incubation caused a significant increase in MCP1 mRNA expression in CD14+ monocytes compared with the control group (*P* < 0.05) (**Figure 3A**). We further examined the effect of LDL on histone acetylation and methylation in the *MCP1* promoter region in CD14+ monocytes. ChIP-qPCR results showed that LDL significantly increased histone H3 and H4 acetylation levels and reduced the level of H3K9 tri-methylation in the promoter region of the *MCP1* gene (**Figures 3B–D**), meanwhile, reduced the recruitments of RFX1, HDAC1 and SUV39H1 in the promoter region of the *MCP1* gene in CD14+ monocytes compared with negative control (**Figures 3E–G**).

### Knockdown of Regulatory Factor X1 Caused the Changes of Histone Modifications in Monocyte Chemoattractant Protein-1 Promoter

**Figure 4:**
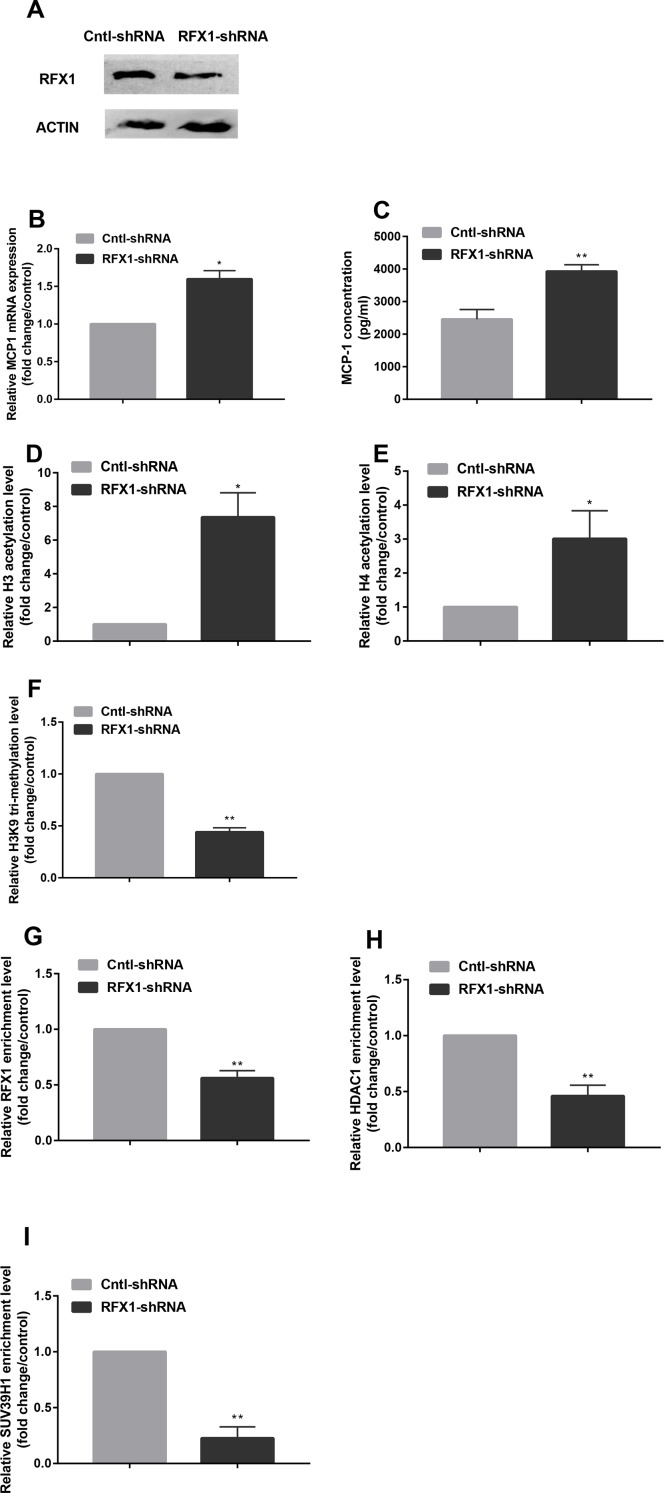
Knockdown of RFX1 caused the changes of histone modifications in *MCP1* promoter. **(A)** The RFX1 protein expression was determined by western blotting in CD14+ monocytes transfected with RFX1-shRNA or Cntl-shRNA. **(B–C)** The relative MCP1 mRNA and protein expression were detected respectively by RT-qPCR and ELISA in CD14+ monocytes transfected with RFX1-shRNA or Cntl-shRNA. **(D–F)** The fold changes of relative H3 **(D)** and H4 **(E)** acetylation levels and H3K9 tri-methylation **(F)** level in CD14+ monocytes transfected with RFX1-shRNA or Cntl-shRNA was determined by ChIP-qPCR. **(G–I)** The fold changes of relative recruitments of RFX1 **(G)**, HDAC1 **(H)** and SUV39H1 **(I)** in the promoter region of the *MCP1* gene were detected by ChIP-qPCR in CD14+ monocytes transfected with RFX1-shRNA or Cntl-shRNA. All values are the average of at least three biological replicates, and the data shown are the mean ± SEM. *P 0.05 and **P 0.01 compared with the control group.

To further investigate the effect of RFX1 knockdown on histone modifications of *MCP1* promoter region by regulating the enrichments of related epigenetic enzymes, we transfected RFX1-shRNA lentiviral vector (RFX1-shRNA) or negative control vector (Cntl-shRNA) into CD14+ monocytes, respectively. As shown in **Figure 4A**, the RFX1 protein expression in CD14+ monocytes transfected with RFX1-shRNA was significantly lower than that in Cntl-shRNA group. In addition, the mRNA level and supernatant concentration of MCP1 in CD14+ monocytes transfected with RFX1-shRNA was obviously increased compared with that in the negative control (**Figures 4B, C**). H3 and H4 acetylation levels in *MCP1* promoter region were increased, but H3K9 tri-methylation level was decreased in CD14+ monocytes transfected with RFX1-shRNA compare with the Cntl-shRNA (**Figures 4D–F**). Compared to Cntl-shRNA group, the enrichments of RFX1, HDAC1 and SUV39H1 in the *MCP1* gene promoter region were significantly decreased in CD14+ monocytes transfected with RFX1-shRNA (**Figures 4G–I**).

### Overexpression of Regulatory Factor X1 Reversed Histone Modifications in Monocyte Chemoattractant Protein-1 Promoter Induced by Low-Density Lipoprotein in CD14+ Monocytes

**Figure 5:**
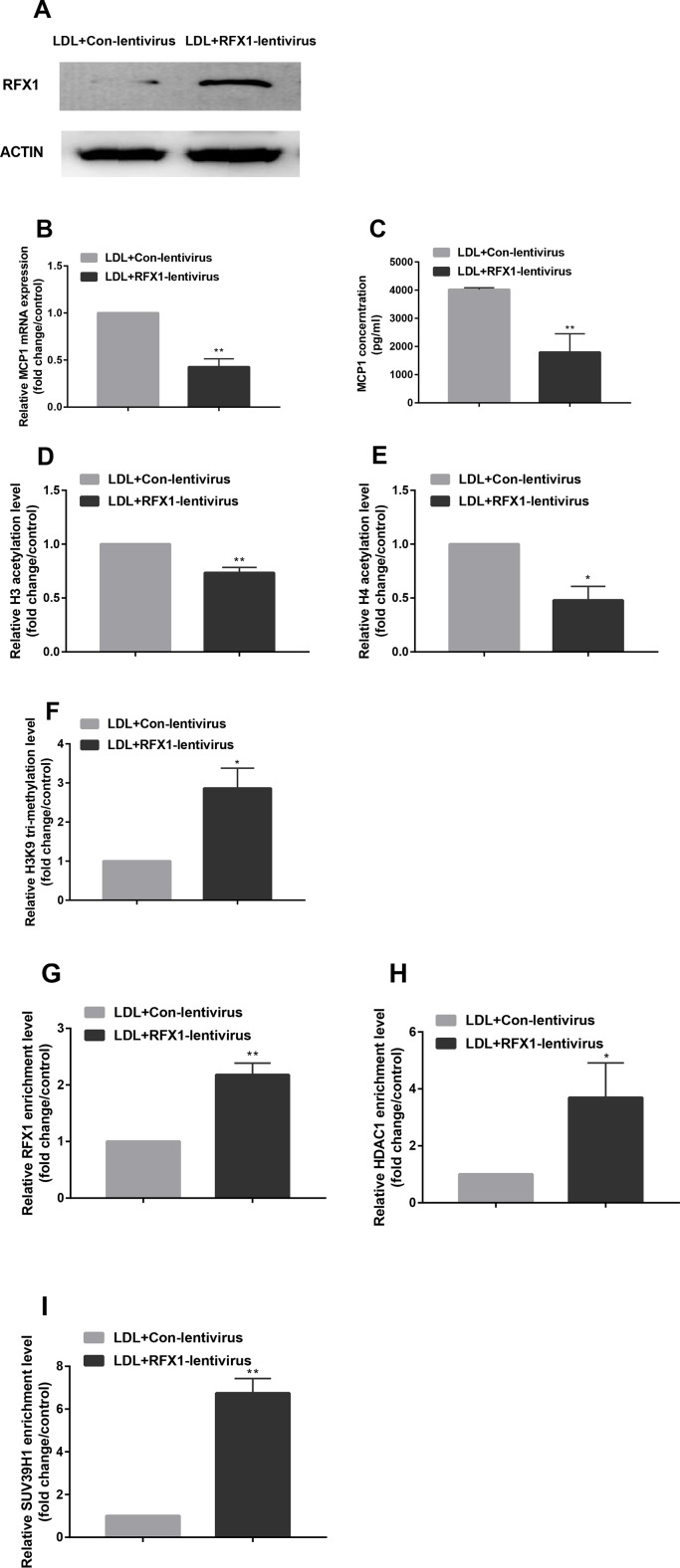
Overexpression of RFX1 reversed histone modifications in *MCP1* promoter induced by LDL in CD14+ monocytes. **(A)** The RFX1 protein expression level was determined by western blotting in LDL-treated CD14+ monocytes transfected with RFX1-lentivirus or Cntl-lentivirus. **(B**–**C)** Relative MCP1 mRNA and protein expression levels were detected respectively by RT-qPCR and ELISA in LDL-treated CD14+ monocytes transfected with RFX1-lentivirus or Cntl-lentivirus. **(D**–**F)** The fold changes of relative H3 **(D)** and H4 **(E)** acetylation levels and H3K9 tri-methylation level **(F)** were detected by ChIP-qPCR in LDL-treated CD14+ monocytes. **(G–I)** The fold changes of relative recruitment levels of RFX1 **(G)**, HDAC1 **(H)** and HUV39H1 **(I)** in the promoter region of the *MCP1* gene were determined by ChIP-qPCR in LDL-treated CD14+ monocytes. All values are the average of at least 3 biological replicates, and the data shown are the mean ± SEM. *P 0.05 and **P 0.01 compared with the control group.

To investigate whether overexpression of RFX1 could reverse the histone modification status of the promoter region of the *MCP1* gene and thereby regulate the expression level of MCP1, we transfected an RFX1 lentivirus expression vector (RFX1-lentivirus) or empty lentivirus (Cntl-lentivirus) into CD14+ monocytes. As shown in **Figure 5A**, the RFX1 protein expression in LDL-treated CD14+ monocytes transfected with RFX1-lentivirus was significantly higher than that in the control group. Moreover, the mRNA level and supernatant concentrations of MCP1 in LDL-incubated CD14+ monocytes transfected with RFX1-lentivirus were obviously downregulated compared with Cntl-lentivirus control (**Figures 5B, C**). Compared to the Cntl-lentivirus group, the histone H3 and H4 acetylation of the *MCP1* gene promoter region was significantly decreased, but the H3K9 tri-methylation level was increased in the LDL-incubated CD14+ monocytes transfected with RFX1-lentivirus (**Figures 5D–F**). The recruitments of RFX1, HDAC1 and SUV39H1 in the promoter region of the *MCP1* gene were significantly increased in LDL-incubated CD14+ monocytes transfected with RFX1-lentivirus compared with those transfected with Cntl-lentivirus (**Figures 5G–I**).

## Discussion

MCP1 can strongly affect the chemotactic of monocytes and increase the adhesion and chemotaxis of monocytes, leading to monocyte activation (Albelda et al., 1994). Early studies in rabbits with AS showed that MCP1 expression was increased in atherosclerotic areas (Yl Herttuala et al., 1991). Monocyte adhesion is dramatically reduced when TNFα-activated primary human pulmonary artery endothelial cell-derived MCP1 is neutralized or inhibited (Maus et al., 2002). Research on human carotid endarterectomy showed that the MCP1 mRNA level detected by *in situ* hybridization was higher in organizing thrombi and in macrophage-rich areas than in normal arteries, which suggested an effect of MCP1 on mediating monocyte infiltration into artery wall (Nelken et al., 1991). In this study, we also found that the expression level of MCP1 in the plasma of CAD patients and MCP1 mRNA expression in CD14+ monocytes from CAD patients were significantly higher than in non-CAD controls. In addition, we examined the DNA methylation and histone acetylation of the *MCP1* promoter region in CD14+ monocytes. However, pyrosequencing experiments showed that the methylation levels of the *MCP1* promoter region of CD14+ monocytes were not different between CAD and non-CAD patients. We speculate that DNA methylation is not the main reason for MCP1 overexpression in CD14+ monocytes from CAD patients. Furthermore, we detected histone tri-methylation and found that histone H3K9 tri-methylation levels in the *MCP1* promoter region of CD14+ monocytes from CAD patients were significantly lower compared with those in the control group.

Aberrant MCP1 expression is closely related to the state of epigenetic modification in the *MCP1* gene promoter region. In the hyperhomocysteinemia model mice, hypomethylation of the *MCP1* gene promoter region in blood monocytes promotes MCP1 expression (Wang et al., 2013). In a mouse model of type 2 diabetes, correlation analysis showed that body mass index, triglyceride levels, and blood glucose levels all affected the methylation level of the promoter region of the *MCP1* gene, which modulated the MCP1 expression level in plasma (Liu et al., 2012). A study on rheumatoid arthritis also showed that incubating THP-1 with TNF-α inhibitors can suppress the *MCP1* gene activation by reducing the recruitment of histone acetyltransferases in the promoter region of the *MCP1* gene (Lin et al., 2017). Recently, Cui et al. reported that Folic Acid could reduce MCP1 expression in the mouse aorta by raising the methylation level of *MCP1* promoter (Cui et al., 2017). Li et al. found that microRNA-124 also can regulate MCP1 expression (Li et al., 2018). All of the above studies suggest that epigenetic modification may play an important role in MCP1 overexpression. Our previous research indicated that the H3K4 and H3K27 tri-methylation levels of the *MCP1* promoter in CAD CD14+ monocytes were not different from non-CAD patients, but the H3 and H4 acetylation of the *MCP1* promoter was increased in CD14+ monocytes from CAD patients (Xiao et al., 2018). In the present study, we further found that there was no difference in DNA methylation levels of CD14+ monocyte from CAD and non-CAD patients. But the H3K9 tri-methylation of *MCP1* promoter in CD14+ monocytes from CAD patients was significantly decreased compared with non-CAD patients (**Figure 1D**). Based on a previous study and this study, we speculate that histone H3 acetylation and H3K9 trimethylation of *MCP1* promoter region rather than DNA methylation contributed to the overexpression of MCP1 in CD14+ monocytes of CAD patients.

High level of LDL is a risk factor for CAD patients, which contributes to the initiation and development of AS (Badimon and Vilahur, 2012). Our research showed that LDL incubation caused significant upregulation of MCP1 mRNA expression in CD14+ monocytes. However, the specific mechanism by which LDL upregulates MCP1 expression is unclear. Studies have shown that ox-LDL stimulation increases H3K4 tri-methylation in the promoter region of several atherogenic genes, including *MCP1*, in human monocytes and facilitates the expression of related proteins (Siroon et al., 2014). We found that compared with the control group, the levels of histone H3 and H4 acetylation in the promoter region of the *MCP1* gene in CD14+ monocytes treated with LDL were significantly increased, and the level of H3K9 tri-methylation was significantly decreased. Therefore, we concluded that LDL caused MCP1 overexpression by altering the histone modification status of the *MCP1* promoter region.

RFX1 contains a C-terminal region that overlaps with the dimeric domain and an N-terminal activation region that can inhibit or activate the transcription of target genes. It has been reported that the expression of RFX1 is decreased in the CD4+ T cells of lupus patients (Zhao et al., 2010a). In addition, RFX1 expression is lower in some types of cancer such as esophageal adenocarcinoma and hepatocellular carcinoma (Watts et al., 2011; Liu et al., 2018). These studies showed that RFX1 exerted a protective effect that inhibited the development of tumors and autoimmune-related diseases such as SLE. Our previous study also showed that RFX1 mRNA and protein expression levels were significantly reduced in CD14+ monocytes from CAD patients and LDL-treated CD14+ monocytes (Du et al., 2019). In addition, RFX1 directly downregulates CD44 expression in glioblastoma, which promotes the survival, proliferation, and invasion of glioblastoma cells (Feng, C. et al., 2014). RFX1 also negatively regulates FGF1 gene expression, which plays an important role in cell growth, proliferation and neurogenesis (Yi-Chao et al., 2010). In peripheral blood CD4+ T cells, RFX1 interacts with HDAC1, DNMT1 and SUV39H1 to affect the histone H3 and H4 acetylation and H3K9 tri-methylation levels of *CD70* and *CD11a* promoter regions (Zhao et al., 2010a; Zhao et al., 2010b; Zhao et al., 2018). Our previous research indicated that RFX1 contributed to the overexpression of TLR4 and the activation of CD14+ monocytes in CAD patients by altering DNA methylation and histone modifications in the *TLR4* promoter region (Du et al., 2019). Therefore, we determined whether RFX1 binds directly to the *MCP1* promoter region. The ChIP assay confirmed that the *MCP1* gene promoter region includes a binding target sequence for RFX1. We then investigated whether RFX1 expression downregulation influences the recruitments of these epigenetically modified enzymes in the *MCP1* promoter region. Our results showed that the binding level of RFX1 in the *MCP1* gene promoter in CAD patients was reduced significantly, which decreased the enrichments of HDAC1 and SUV39H1, therefore leading to overexpression of MCP1 by downregulating histone H3 and H4 acetylation and upregulating H3K9 tri-methylation in the promoter region of the *MCP1* gene. To further validate the regulation of RFX1 on the *MCP1* promoter region, we overexpressed or interfered with RFX1 in CD14+ monocytes. The results indicated that RFX1 was involved in regulating the recruitments of related epigenetic modification enzymes and histone modification status in the *MCP1* promoter region in CD14+ monocytes, which further suggested that RFX1 may be a protective transcription factor for CAD. In our study, we observed that histone modifications in MCP1 promoter, but not DNA methylation, were changed in CAD patients compared with non-CAD patients. The results indicated that DNA methylation is not involved in regulating MCP1 overexpression in CD14+ monocytes of CAD patients. Although MCP1 is a target gene of transcription factor RFX1, MCP1 expression also may be regulated by other transcription factors. Therefore, we speculate that there may exist other regulatory mechanisms influencing the DNA methylation status of MCP1 promoter in CD14+ monocytes of CAD patients, which lead to no significant difference in DNA methylation level of MCP1 promoter between CAD patients and non-CAD patients.

In summary, this study investigated the mechanism of MCP1 overexpression in CD14+ monocytes from CAD patients, and found that RFX1 plays a vital role in this process. The downregulated expression of RFX1 decreased recruitments of HDAC1 and SUV39H1 in the promoter region of *MCP1* gene to induce the increased histone acetylation and the decreased H3K9 tri-methylation of *MCP1* promoter. This finding demonstrated further the role of transcription factor RFX1 in the pathogenesis of CAD, suggesting an important target for CAD treatment.

## Data Availability Statement

The datasets generated for this study are available on request to the corresponding author.

## Ethics Statement

This study was approved by the Ethics Committee of the Third Xiangya Hospital of Central South University, and written informed consent was obtained from all subjects.

## Author Contributions

MZ and SJ designed the research and revised the manuscript. SY and SJ conducted the research. YC organized and processed the biospecimens. PD, KG, and BY analyzed the data. RG obtained the clinical data. SY wrote the main manuscript. MZ had primary responsibility for the final content. All authors agree to be accountable for the content of the work.

## Funding

This work was supported by grants from the National Natural Science Foundation of China (No. 81370392) and the Fundamental Research Funds for the Central Universities of Central South University (2018zzts946).

## Conflict of Interest

The authors declare that the research was conducted in the absence of any commercial or financial relationships that could be construed as a potential conflict of interest.

## References

[B1] Albelda S. M. Smith C. W. Ward P. A. (1994). Adhesion molecules and inflammatory injury. Faseb J. 8 (8), 504–512. 10.1096/fasebj.8.8.8181668 8181668

[B2] Annika L. Derek M. Frank B. Tonatiuh M. (2007). Innate immunity and inflammation–New frontiers in comparative cardiovascular pathology. Cardiovasc. Res. 73 (1), 26–36. 10.1016/j.cardiores.2006.08.009. 17010957

[B3] Badimon L. Vilahur G. (2012). LDL-cholesterol versus HDL-cholesterol in the atherosclerotic plaque: inflammatory resolution versus thrombotic chaos. Ann. N. Y. Acad. Sci. 1254, 18–32. 10.1111/j.1749-6632.2012.06480.x 22548566

[B4] Boisvert W. A. Curnss L. K. Terkeltaub R. A. (2000). Interleukin-8 and its receptor CXCR2 in atherosclerosis. Immunologic Res. 21 (2-3), 129–137. 10.1385/IR:21:2-3:129 10852110

[B5] Chen K. C. Liao Y. C. Hsieh I. C. Wang Y. S. Hu C. Y. Juo S. H. (2012). OxLDL causes both epigenetic modification and signaling regulation on the microRNA-29b gene: novel mechanisms for cardiovascular diseases. J. Mol. Cell. Cardiol. 52 (3), 587–595. 10.1016/j.yjmcc.2011.12.005 22226905

[B6] Cheng K. Sun H. Zhang M. Shen L. Biochemistry D. O. (2016). Overexpression of lentivirus RFXI and its inhibitory effect on proliferation of glioblastoma cells. J. Central South University 41 (11), 1117–1123. 10.11817/j.issn.1672-7347.2016.11.001 27932754

[B7] Chistiakov D. A. Bobryshev Y. V. Orekhov A. N. (2016). Macrophage-mediated cholesterol handling in atherosclerosis. J. Cell. Mol. Med. 20 (1), 17–28. 10.1111/jcmm.12689 26493158 PMC4717859

[B8] Cui S. Li W. Lv X. Wang P. Gao Y. Huang G. (2017). Folic acid supplementation delays atherosclerotic lesion development by modulating MCP1 and VEGF DNA methylation levels in vivo and in vitro. Int. J. Mol. Sci. 18 (5), 990–1008. 10.3390/ijms18050990. PMC5454903 28475147

[B9] Cushing S. D. Fogelman A. M. (1992). Monocytes may amplify their recruitment into inflammatory lesions by inducing monocyte chemotactic protein. Arterioscler Thromb. 12 (1), 78–82. 10.1161/01.ATV.12.1.78 1731861

[B10] Du H. P. Li J. You S. J. Wang Y. L. Wang F. Cao Y. J. (2016). DNA methylation in cystathionine-Î³-lyase (CSE) gene promoter induced by ox-LDL in macrophages and in apoE knockout mice. Biochem. Biophys. Res. Commun. 469 (3), 776–782. 10.1016/j.bbrc.2015.11.132 26692478

[B11] Du P. Gao K. Cao Y. Yang S. Wang Y. Guo R. (2019). RFX1 downregulation contributes to TLR4 overexpression in CD14(+) monocytes *via* epigenetic mechanisms in coronary artery disease. Clin. Epigenet. 11 (1), 44. 10.1186/s13148-019-0646-9 PMC6413463 30857550

[B12] Emery P. Durand B. Mach B. Reith W. (1996). RFX proteins, a novel family of DNA binding proteins conserved in the eukaryotic kingdom. Nucleic Acids Res. 24 (5), 803–807. 10.1093/nar/24.5.803 8600444 PMC145730

[B13] Escate R. Padro T. Badimon L. (2016). LDL accelerates monocyte to macrophage differentiation: Effects on adhesion and anoikis. Atherosclerosis 246, 177–186. 10.1016/j.atherosclerosis.2016.01.002 26800307

[B14] Feng C. Zhang Y. Yin J. Li J. (2014a). Regulatory factor X1 is a new tumor suppressive transcription factor that acts via direct downregulation of CD44 in glioblastoma. Neuro Oncol. 16 (8), 1078–1085. 10.1093/neuonc/nou010 24526308 PMC4096175

[B15] Feng Y. Cai Z. R. Tang Y. Hu G. Lu J. He D. (2014b). TLR4/NF-κB signaling pathway-mediated and oxLDL-induced up-regulation of LOX-1, MCP-1, and VCAM-1 expressions in human umbilical vein endothelial cells. Genet. Mol. Res. Gmr 13 (1), 680–695. 10.4238/2014.January.28.13 24615033

[B16] Ghattas A. Griffiths H. R. Devitt A. Lip G. Y. H. Shantsila E. (2013). Monocytes in coronary artery disease and atherosclerosis. J. Am. College Cardiol. 62 (17), 1541–1551. 10.1016/j.jacc.2013.07.043 23973684

[B17] Gosling J. Slaymaker S. Gu L. Tseng S. Zlot C. H. Young S. G. (1999). MCP-1 deficiency reduces susceptibility to atherosclerosis in mice that overexpress human apolipoprotein B. J. Clin. Invest. 103 (6), 773–778. 10.1172/JCI5624 10079097 PMC408147

[B18] Greißel A. Culmes M. Napieralski R. Wagner E. Gebhard H. Schmitt M. (2015). Alternation of histone and DNA methylation in human atherosclerotic carotid plaques. Thrombosis Haemostasis 114 (02), 390–402. 10.1160/TH14-10-0852 25993995

[B19] Hohensinner P. J. Niessner A. Huber K. Weyand C. M. Wojta J. (2011). Inflammation and Cardiac Outcome. Curr. Opin. Infect. Dis. 24 (3), 259–264. 10.1097/QCO.0b013e328344f50f 21378564 PMC4497511

[B20] Katan-Khaykovich Y. Shaul Y. (1998). RFX1, a single DNA-binding protein with a split dimerization domain, generates alternative complexes. J. Biol. Chem. 273 (38), 24504–24512. 10.1074/jbc.273.38.24504 9733744

[B21] Kolattukudy P. E. Niu J. Inflammation E. R. (2012). Stress Autophagy MCP-1/CCR2 Pathway. Circulation Res. 110 (1), 174. 10.1161/CIRCRESAHA.111.243212 22223213 PMC3265021

[B22] Kuziel W. A. Morgan S. J. Dawson T. C. Griffin S. Smithies O. Ley K. (1997). Severe reduction in leukocyte adhesion and monocyte extravasation in mice deficient in CC chemokine receptor 2. Proc. Natl. Acad. Sci. U.S.A. 94 (22), 12053–12058. 10.1073/pnas.94.22.12053 9342361 PMC23699

[B23] Ley K. Miller Y. I. Hedrick C. C. (2011). Monocyte and macrophage dynamics during atherogenesis. Arterioscler Thromb. Vasc. Biol. 31 (7), 1506–1516. 10.1161/ATVBAHA.110.221127 21677293 PMC3133596

[B24] Li X. Zhang Y. Xu G. Li S. Li H. (2018). miR-124/MCP-1 signaling pathway modulates the protective effect of itraconazole on acute kidney injury in a mouse model of disseminated candidiasis. Int. J. Mol. Med. 41 (6), 3468–3476. 10.3892/ijmm.2018.3564 29568906

[B25] Lin Y. C. Lin Y. C. Huang M. Y. Kuo P. L. Wu C. C. Lee M. S. (2017). Tumor necrosis factor-alpha inhibitors suppress CCL2 chemokine in monocytes via epigenetic modification. Mol. Immunol. 83, 82–91. 10.1016/j.molimm.2017.01.009 28113136

[B26] Liu Z. H. Chen L. L. Deng X. L. Song H. J. Liao Y. F. Zeng T. S. (2012). Methylation status of CpG sites in the MCP-1 promoter is correlated to serum MCP-1 in Type 2 diabetes. J. Endocrinol. Invest. 35 (6), 585–589. 10.3275/7981 21975431

[B27] Liu Y. Jiang P. Wang G. Liu X. Luo S. (2018). Downregulation of RFX1 predicts poor prognosis of patients with small hepatocellular carcinoma. Eur. J. Surg. Oncol. 44 (7), S0748798318310230. 10.1016/j.ejso.2018.04.017. 29764705

[B28] Maus U. Henning S. Wenschuh H. Mayer K. Seeger W. Lohmeyer J. (2002). Role of endothelial MCP-1 in monocyte adhesion to inflamed human endothelium under physiological flow. Am. J. Physiol. Heart Circulatory Physiol. 283 (6), H2584. 10.1152/ajpheart.00349.2002 12388329

[B29] Nelken N. A. Coughlin S. R. Gordon D. Wilcox J. N. (1991). Monocyte chemoattractant protein-1 in human atheromatous plaques. J. Clin. Invest. 88 (4), 1121–1127. 10.1172/JCI115411 1843454 PMC295565

[B30] Ohashi Y. Ueda M. T. Kawakami Y. Toda M. (2004). Identification of an epigenetically silenced gene, RFX1, in human glioma cells using restriction landmark genomic scanning. Oncogene 23 (47), 7772–7779. 10.1038/sj.onc.1208058 15334059

[B31] Peng H. Gao D. Wei Z. Liu S. Yang S. Li X. (2015). Puerarin suppresses high glucose-induced MCP-1 expression via modulating histone methylation in cultured endothelial cells. Life Sci. 130, 103–107. 10.1016/j.lfs.2015.02.022 25817234

[B32] Siroon B. Jessica Q. Joosten L. A. B. Meer J. W. M. V. Netea M. G. Riksen N. P. (2014). Oxidized low-density lipoprotein induces long-term proinflammatory cytokine production and foam cell formation via epigenetic reprogramming of monocytes. Arteriosclerosis Thrombosis Vascular Biol. 34 (8), 1731–1738. 10.1161/ATVBAHA.114.303887 24903093

[B33] Tenjin N. Shinji K. Yuya Y. Tsutomu H. Fumiyoshi T. Makoto S. (2014). Elevated small dense low-density lipoprotein cholesterol as a predictor for future cardiovascular events in patients with stable coronary artery disease. J. Atherosclerosis Thrombosis 21 (8), 755–767. 10.5551/jat.23465 24717762

[B34] Wang J. Jiang Y. Yang A. Sun W. Ma C. Ma S. (2013). Hyperhomocysteinemia-Induced Monocyte Chemoattractant Protein-1 Promoter DNA Methylation by Nuclear Factor-κB/DNA Methyltransferase 1 in Apolipoprotein E–Deficient Mice. Bioresearch Open Access 2 (2), 118–127. 10.1089/biores.2012.0300 23593564 PMC3620495

[B35] Watts J. A. Chaolin Z. Klein-Szanto A. J. Kormish J. D. Jian F. Zhang M. Q. (2011). Study of FoxA pioneer factor at silent genes reveals Rfx-repressed enhancer at Cdx2 and a potential indicator of esophageal adenocarcinoma development. PLoS Genet. 7 (9), e1002277. 10.1371/journal.pgen.1002277 21935353 PMC3174211

[B36] Xiao L. I. Cao Y. Wang Y. Lai X. Gao K. Q. Du P. (2018). Aberrant histone modifications of global histone and MCP-1 promoter in CD14(+) monocytes from patients with coronary artery disease. Pharmazie 73 (4), 202–206. 10.1691/ph.2018.7342 29609686

[B37] Yi-Chao H. Wei-Chih L. Chien-Yu K. Ing-Ming C. (2010). Regulation of FGF1 gene promoter through transcription factor RFX1. J. Biol. Chem. 285 (18), 13885–13895. 10.1074/jbc.M109.081463 20189986 PMC2859551

[B38] Yl Herttuala S. Lipton B. A. Rosenfeld M. E. Rkioja T. Yoshimura T. Leonard E. J. (1991). Expression of monocyte chemoattractant protein 1 in macrophage-rich areas of human and rabbit atherosclerotic lesions. Proc. Natl. Acad. Sci. U. S. A. 88 (12), 5252–5256. 10.1073/pnas.88.12.5252 2052604 PMC51850

[B39] Zhang E. Wu Y. (2013). MicroRNAs: important modulators of oxLDL-mediated signaling in atherosclerosis. J. Atherosclerosis Thrombosis 20 (3), 215–227. 10.5551/jat.15180 23064493

[B40] Zhao M. Sun Y. Gao F. Wu X. Tang J. Yin H. (2010a). Epigenetics and SLE: RFX1 downregulation causes CD11a and CD70 overexpression by altering epigenetic modifications in lupus CD4+ T cells. J. Autoimmunity 35 (1), 58–69. 10.1016/j.jaut.2010.02.002 20223637

[B41] Zhao M. Wu X. Zhang Q. Luo S. Liang G. Su Y. (2010b). RFX1 regulates CD70 and CD11a expression in lupus T cells by recruiting the histone methyltransferase SUV39H1. Arthritis Res. Ther. 12 (6), R227. 10.1186/ar3214 21192791 PMC3046540

[B42] Zhao M. Tan Y. Peng Q. Huang C. Guo Y. Liang G. (2018). IL-6/STAT3 pathway induced deficiency of RFX1 contributes to Th17-dependent autoimmune diseases via epigenetic regulation. Nat. Commun. 9 (1), 583. 10.1038/s41467-018-02890-0 29422534 PMC5805701

[B43] Zidar D. A. Juchnowski S. Ferrari B. Clagett B. Pilch-Cooper H. A. Rose S. (2015). Oxidized LDL Levels Are Increased in HIV Infection and May Drive Monocyte Activation. J. Acquir. Immune Defic. Syndr. 69 (2), 154–160. 10.1097/QAI.0000000000000566 25647528 PMC4446174

