# Supplementary material for: Regulatory Factor X1 Downregulation Contributes to Monocyte Chemoattractant Protein-1 Overexpression in CD14+ Monocytes via Epigenetic Mechanisms in Coronary Heart Disease
Source: Front Genet. 2019 Nov 1;10:1098. doi: 10.3389/fgene.2019.01098 (PMC6838212; doi:10.3389/fgene.2019.01098)
Supplement: Supplementary file 1 [file Table_1.docx]

Supplementary Material

# Supplementary Table 1. General information of CAD patients and non-CAD subjects.

|  | CAD  (n = 42) | Non-CAD  （n = 27） | *P* |
| --- | --- | --- | --- |
| Gender (M/F) | (19/23) | (16/11) | 0.382 |
| Age | 62.52 ± 9.80 | 60.52 ± 8.72 | 0.345 |
| Systolic blood pressure (mmHg) | 134.83 ± 19.87 | 134.68 ± 17.16 | 0.972 |
| Diastolic blood pressure (mmHg) | 80.15 ± 9.63 | 81.53 ± 9.28 | 0.516 |
| Creatinine (μmol/L) | 71.48 ± 16.90 | 79.27 ± 18.49 | 0.044 |
| Heart rate (bpm) | 73.96 ± 9.72 | 72.16 ± 6.90 | 0.374 |
| Triglyceride (mmol/L) | 1.73 ± 1.02 | 1.45 ± 0.54 | 0.171 |
| Cholesterol (mmol/L) | 4.65 ± 0.96 | 4.38 ± 0.92 | 0.204 |
| Low-density lipoprotein (mmol/L) | 3.12 ± 4.57 | 2.42 ± 0.64 | 0.433 |
| High density lipoprotein (mmol/L) | 1.27 ± 0.26 | 1.14 ± 0.26 | 0.027* |
| Alanine aminotransferase (U/L) | 30.96 ± 24.08 | 28.19 ± 15.10 | 0.576 |
| Diabetes (%) | 14 (33.3) | 5 (18.5) | 0.114 |
| Hypertension (%) | 25 (59.5) | 14 (51.9) | 0.558 |

# **Supplementary Table 2**. List of primers applied for RT-qPCR.

| Gene | Primer | Sequence（5’-3’） |
| --- | --- | --- |
| RFX1 | Forward | 5’-GATCCAAGGCGGCTACAT-3’ |
|  | Reverse | 5’-CAGCCGTCTCATAGTTGTCC-3’ |
| MCP1 | Forward | 5’-TACAAAATCCCCGACAACCTCC-3’ |
|  | Reverse | 5’-GCTGCCTAAATGCCTCAGGG-3’ |
| β-actin | Forward | 5’-GCACCACACCTTCTACAATGAGC-3’ |
|  | Reverse | 5’-GGATAGCACAGCCTGGATAGCAAC-3’ |

# Supplementary Table 3. List of primers applied for Pyrophosphate Sequencing.

| Gene | Primer | Sequence |
| --- | --- | --- |
| MCP1-1 | Forward | 5’- GAGAATTGGATGTTTTTGGGTTAGT -3’ |
|  | Reverse | 5’-ACTCAACAAATTTAACAACCCACTTAT-3’ |
|  | Sequencing | 5’-GTTGTTTTTGTTTTTTATTGAAAG-3’ |
| MCP1-2 | Forward | 5’-GGAGGGATTTTTTATGAGTGATAAG-3’ |
|  | Reverse | 5’- TCCCCATTTACTCATTTAATCTCAACAATA-3’ |
|  | Sequencing | 5’-AAAGGGAAGTAGGGG-3’ |

# Supplementary Table 4. List of primers applied for ChIP-qPCR.

| Gene | Primer | Sequence（5’-3’） |
| --- | --- | --- |
| MCP1-1 | Forward | 5’-CGCTTCCTTCCTTTTCTGCA-3’ |
|  | Reverse | 5’-TGCCATTAAGCCCAGACTGA-3’ |
| MCP1-2 | Forward | 5’-CTGTGAACCCCAAATCCAGC-3’ |
|  | Reverse | 5’-ACCCTGATCCCCAAACTCTG-3’ |
